# Advances in Metabolic Profiling and Pharmacokinetics of Herbal Medicinal Products

**DOI:** 10.1155/2019/5190972

**Published:** 2019-05-06

**Authors:** Xinguang Liu, Yong Ai, Jiang Ma

**Affiliations:** ^1^Collaborative Innovation Center for Respiratory Disease Diagnosis and Treatment & Chinese Medicine Development of Henan Province, Henan University of Chinese Medicine, Zhengzhou, Henan, China; ^2^Institute of Integrative Medicine, Dalian Medical University, Dalian, Liaoning, China; ^3^University of Maryland, Baltimore, USA; ^4^School of Biomedical Sciences, The Chinese University of Hong Kong, Hong Kong

Herbal medicinal products (HMPs) are expected to be safe and effective. Nevertheless, evidence-based verification of the safety and efficacy of HMPs is still lacking. Understanding the metabolic profile and* in vivo* fate of HMPs has been considered an important issue to identify pharmacologically active or toxic compounds in HMPs or their metabolites for further discovery and development of new drugs or for safety monitoring. These processes include investigation of bioavailability to assess to what degree and how fast compounds are absorbed after drug administration, elucidation of metabolic pathways, and elimination routes and their kinetics, as well as the interactions of HMPs with synthetically derived drug products. Due to the complex chemical composition of HMPs, the absorption, distribution, metabolism, and excretion characteristics of the bioactive or toxic HMPs remain to be further explored. The difficulty lies primarily in the selection of appropriate biomarkers for detection, quantification of trace constituents, identification of potential drug-drug interactions, and the discovery of active constituents based on metabolic results.

 In this special issue, investigators contribute original research articles and review articles that would facilitate the understanding of the basic mechanisms as well as the development of new and promising complementary and alternative strategies for the metabolic profiling and pharmacokinetics of HMPs.

Integrating pharmacokinetics is a crucial tool to identify the therapeutic agent. The research article by C. Zhang et al. elucidated the pharmacokinetic profiles and disposition kinetics of the administered and generated stereoisomers in the brain and cerebrospinal fluid (CSF) after oral administration of Isorhynchophylline (IRN) and Rhynchophylline (RN). The results demonstrated that, after oral administration, RN showed significantly higher systemic exposure and disposition in the brain and CSF than IRN, indicating that RN would be more appropriate to be developed as a potential therapeutic agent for the treatment of AD.

In technology, ultrahigh-performance liquid chromatography tandem mass spectrometry (UPLC-MS/MS) is still the most popular tool for the pharmacokinetics study of HMPs. The research article by Y. Fu et al. established a simple, sensitive, and reliable UPLC-MS/MS method for quantifying pinosylvin in rat plasma, urine, feces, and various tissues. Nine metabolites of pinosylvin were found in plasma, and glucuronidation, hydroxylation, and methylation proved to be its main metabolic pathways.

There are many factors affecting the pharmacokinetics of HMPs, among which herb-drug interaction has been frequently studied. The research article by D. Yim et al. examined the selective interaction of* Sophora flavescens* extract with cytochrome P450 (CYP) isoforms in human liver microsomes. The result showed that* S. flavescens *and its prenylated flavonoids caused significant herb-drug interactions when coadministered or incubated with substrates of CYP2B6, CYP2C8, CYP2C9, and CYP3A4. The research article by N. Temyingyong et al. determined the effect of short-course oral ciprofloxacin on isoflavone pharmacokinetics in healthy postmenopausal women. The results showed that ciprofloxacin administration significantly reduced the absorption of the aglycones of genistein and daidzein.

Drug formulation and administration route also significantly affect the pharmacokinetics of HMPs. The research article by P. Smyk et al. elucidated the influence of propolis on ion channels, channels location in cell membrane, and electrical resistance of skin. In this work, a model skin of Ussing chamber proved to be useful for studying the effect of chemicals on transepidermal ion transport.

Finally, in addition to herb-drug interaction, the review article by S. Sun et al. summarized other important factors that affect the clinical practice of HMPs, such as herb pretreatment, herb-herb interactions, pathological status, gender, age of patients, and chemical and physical modification of certain ingredients.

Thus, this special issue included different aspects related to the recent advances in metabolic profiling and pharmacokinetics of HMPs, based on various* in vitro* and* in vivo* studies as well as the literature review related to the objective of this special issue.

